# Characterization of a thermostable β-glucosidase from *Aspergillus fumigatus *Z5, and its functional expression in *Pichia pastoris *X33

**DOI:** 10.1186/1475-2859-11-25

**Published:** 2012-02-17

**Authors:** Dongyang Liu, Ruifu Zhang, Xingming Yang, Zhenhua Zhang, Song Song, Youzhi Miao, Qirong Shen

**Affiliations:** 1 Jiangsu Key Lab for Organic Solid Waste Utilization, Nanjing Agricultural University, Nanjing 210095, China; 2 College of Resources and Environmental Science, Nanjing Agricultural University, Nanjing, China

## Abstract

**Background:**

Recently, the increased demand of energy has strongly stimulated the research on the conversion of lignocellulosic biomass into reducing sugars for the subsequent production, and β-glucosidases have been the focus because of their important roles in a variety fundamental biological processes and the synthesis of useful β-glucosides. Although the β-glucosidases of different sources have been investigated, the amount of β-glucosidases are insufficient for effective conversion of cellulose. The goal of this work was to search for new resources of β-glucosidases, which was thermostable and with high catalytic efficiency.

**Results:**

In this study, a thermostable native β-glucosidase (nBgl3), which is secreted by the lignocellulose-decomposing fungus *Aspergillus fumigatus *Z5, was purified to electrophoretic homogeneity. Internal sequences of nBgl3 were obtained by LC-MS/MS, and its encoding gene, *bgl3*, was cloned based on the peptide sequences obtained from the LC-MS/MS results. *bgl*3 contains an open reading frame (ORF) of 2622 bp and encodes a protein with a predicted molecular weight of 91.47 kDa; amino acid sequence analysis of the deduced protein indicated that nBgl3 is a member of the glycoside hydrolase family 3. A recombinant β-glucosidase (rBgl3) was obtained by the functional expression of *bgl*3 in *Pichia pastoris *X33. Several biochemical properties of purified nBgl3 and rBgl3 were determined - both enzymes showed optimal activity at pH 6.0 and 60°C, and they were stable for a pH range of 4-7 and a temperature range of 50 to 70°C. Of the substrates tested, nBgl3 and rBgl3 displayed the highest activity toward 4-Nitrophenyl-β-D-glucopyranoside (pNPG), with specific activities of 103.5 ± 7.1 and 101.7 ± 5.2 U mg^-1^, respectively. However, these enzymes were inactive toward carboxymethyl cellulose, lactose and xylan.

**Conclusions:**

An native β-glucosidase nBgl3 was purified to electrophoretic homogeneity from the crude extract of *A. fumigatus *Z5. The gene *bgl*3 was cloned based on the internal sequences of nBgl3 obtained from the LC-MS/MS results, and the gene *bgl3 *was expressed in *Pichia pastoris *X33. The results of various biochemical properties of two enzymes including specific activity, pH stability, thermostability, and kinetic properties (Km and Vmax) indicated that they had no significant differences.

## Background

The β-glucosidase enzyme plays important roles and exists in most of the living kingdoms, from simple bacteria to highly complex mammals [1]. β-glucosidase obtained from various sources has been widely used for many applications, such as the enzymatic saccharification of cellulosic materials, the liberation of flavor compounds in fruit juices and wines, and the release of phenolic compounds with antioxidant activity from fruit and vegetable residues [2-4]. The increased need for a considerable β-glucosidase activity, especially in the enzymatic saccharification of cellulose for bioenergy, has strongly stimulated the study of β-glucosidase.

Cellulose, a virtually inexhaustible source of renewable bioenergy, is the most abundant polysaccharide in nature and the major component of plant cell walls [5]. However, without appropriate treatment, a mass of agricultural, industrial and municipal cellulosic wastes has accumulated, resulting in the risk of environmental pollution [6]. Various methods, such as composting, mechanical treatment and chemical treatment, have been applied to treat these cellulosic wastes [7,8]. Ethanol production from lignocellulosic biomass has received much attention due to the immense potential for conversion of renewable biomaterials into biofuels and chemicals [9]. The hydrolysis of cellulose primarily depends on at least three enzymes, including endoglucanases (EGs), cellobiohydrolases (CBHs) and β-glucosidases, which work synergistically to degrade the cellulose [10]. EGs and CBHs can degrade native cellulose synergistically to generate cellobiose, which is a product inhibitor for these enzymes [11]. However, β-glucosidases can scavenge the end product cellobiose by cleaving the β (1-4) linkage to generate D-glucose. Thus, β-glucosidases allow the cellulolytic enzymes to function more efficiently by producing glucose from cellobiose and reducing cellobiose inhibition [12]. Furthermore, when a β-glucosidase was added to lignocellulosic materials, the release of phenolic compounds increased, indicating that cellulose-degrading enzymes may also be involved in the breakdown of polymeric phenolic matrices [13].

Enzyme thermostability is essential during the saccharification step because steam is always used to make the substrates more suitable for enzymatic hydrolysis [14]. Thermostable enzymes can be used simultaneously and directly in the saccharification procedure without a pre-cooling process. Obtaining efficient and thermostable β-glucosidase has become the goal of much research worldwide. Currently, most of the β-glucosidases for industrial applications are secreted by microorganisms, and β-glucosidases from fungi have been extensively studied in some model organisms, such as *Trichoderma reesei *[15] and *Phanerochaete chrysosporium *[16]. The β-glucosidases from some species of *Aspergillus *are also well studied and include the β-glucosidase secreted by *Aspergillus terreus *(EC 3.2.1.21) [17], the β-glucosidase purified from crude cellulase of *Aspergillus niger *[18], and a novel, highly glucose-tolerant β-glucosidase from *Aspergillus oryzae *[19]. However, few researchers, except Rudick & Elbein [20], have focused on β-glucosidase from the thermophilic strain *A. fumigatus*, which can secret thermostable cellulase. The lignocellulose-decomposing fungus *A. fumigatus *Z5 was isolated from compost, and the preliminary results indicated that thermostable β-glucosidase was secreted into the medium when cellulose was used as the sole carbon source.

In this study, a thermostable enzyme native Bgl3 (nBgl3) was purified from a lignocellulose-decomposing fungus, *A. fumigatus *Z5, and the nBgl3-encoding gene was cloned by employing degenerate PCR and High-efficiency thermal asymmetric interlaced PCR (hiTAIL-PCR). Recombinant Bgl3 (rBgl3) was expressed in *P. pastoris *X33 and purified with Ni-NTA Sepharose. The properties of these two enzymes, including substrate specificity and amino acid sequence, indicate that nBgl3 and rBgl3 are β-glucosidases, members of the glycoside hydrolase family 3 (GH3) category of enzymes.

## Results

### Purification and identification of β-glucosidase secreted by *A. fumigatus *Z5

**Figure 1:**
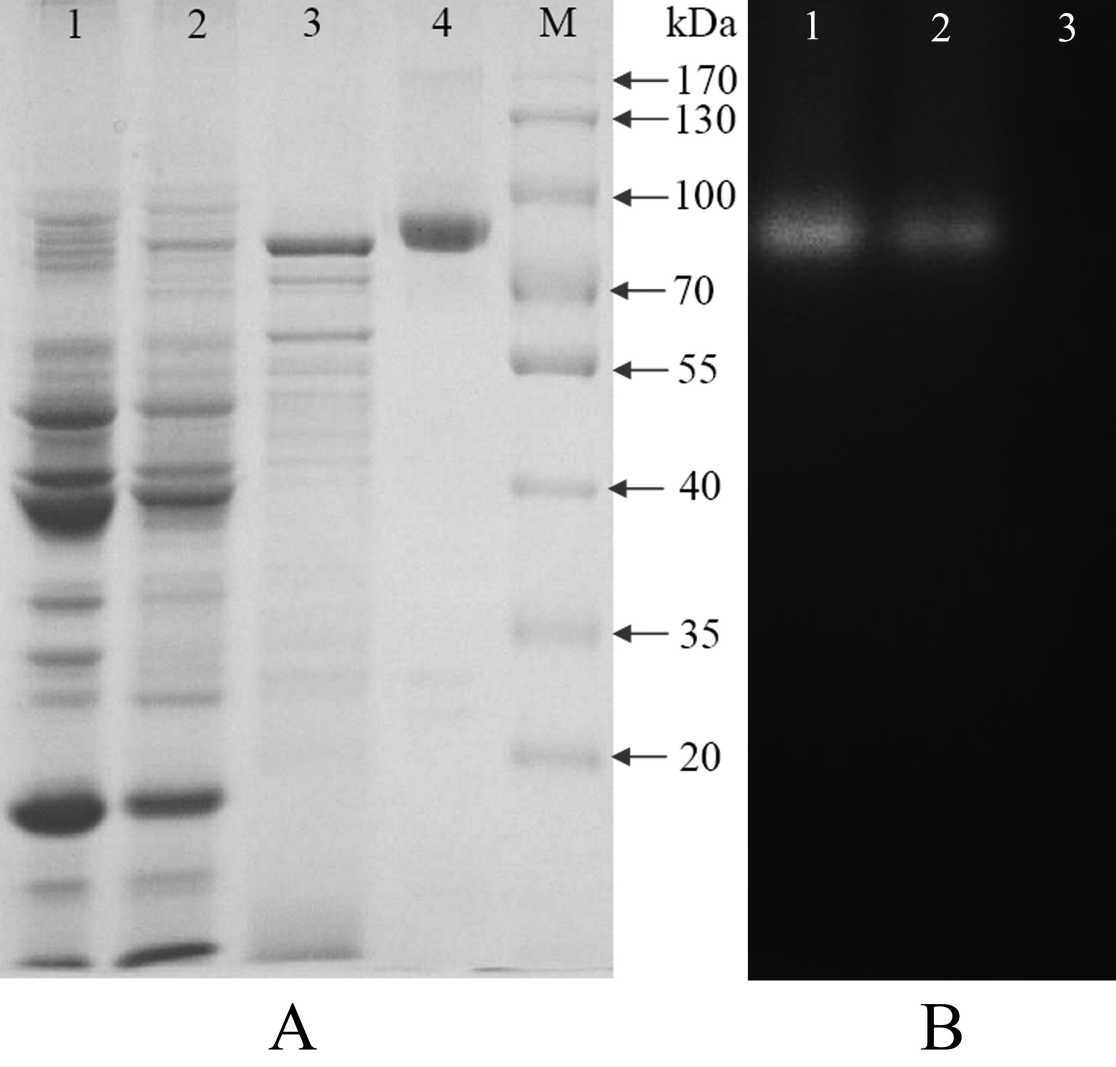
**Purification, SDS-PAGE (A) and native PAGE (B) analysis of the purified native Bgl3 enzyme secreted by *A. fumigatus *Z5**. A, Lane 1: crude enzyme secreted by *A. fumigatus *Z5; Lane 2: fractions after Sephadex G-100 column; Lane 3: fractions after Q-sepharose FF column; Lane 4: protein of purified β-glucosidase after microcrystalline cellulose column; Lane M: molecular weight markers. B, Lane 1: crude enzyme secreted by *A. fumigatus *Z5; Lane 2: purified native Bgl3; lane 3: blank.

An extracellular β-glucosidase (nBgl3) secreted by *A. fumigatus *Z5 was successfully purified using ammonium sulfate precipitation, followed by Q-sepharose FF, Sephadex G-100 and microcrystalline cellulose (MC) column chromatography; the purification steps are summarized in Table 1. Ammonium sulfate precipitation (80% saturation) resulted in a 1.1-fold purification increase, with 92.8% of the enzyme present in the crude extract. The major β-glucosidase peak was eluted with a NaCl gradient (0-0.3 M) during Q-sepharose FF column chromatography. This purification step resulted in a 3.1-fold increase in purification; however, this eluate contained endoglucanase activities. These fractions were loaded onto a Sephadex G-100 column, and this gel filtration step resulted in a 5.1-fold increase in purification. After the MC column chromatography step, the nBgl3 enzyme was purified 85.1-fold with a specific activity of 127.7 U mg^-1^. SDS-PAGE showed a single protein band, and the molecular mass of the purified nBgl3 was estimated to be approximately 92 kDa (Figure 1A). Activity of the purified nBgl3 was detected by native PAGE analysis, and the results indicated that this band was able to hydrolyze MUG and create a clear fluorescent band in the gel after being visualized under UV light at 365 nm (Figure 1B).

**Table 1:** Summary of purification of β-glucosidase secreted by *A. fumigatus *Z5

Purification steps	Total protein (mg)	Total activity (U)	Specific activity (U mg^-1^)	Recovery (%)	Purification (fold)
Crude extract	632.2	941.3	1.5	100	1.0

Ammonium sulphate precipitation	501.9	873.1	1.7	92.8	1.1

Q-sepharose FF (ion exchange)	74.1	351.1	4.7	37.3	3.1

Sephadex G-100 (gel filtration)	24.9	188.4	7.6	20.0	5.1

Microcrystalline cellulose	0.7	89.4	127.7	9.5	85.1

Each value represents the mean of triplicate measurements and varies from the mean by not more than 10%.

The internal amino acid sequence of the purified nBgl3 was obtained by liquid chromatography coupled with tandem mass spectrometry (LC-MS/MS). The MS data analysis was performed by searching related sequences in the NCBI non-redundant (NCBI nr) databases, and two protein hits were obtained. The first hit was a putative β-glucosidase from *A. fumigatus *Af293 (gi|70990956) with a mass of 95093 Da, and 4 peptides were matched to this protein: IPPNFSSWTR, HYILNEQEHFR, DLANWDVEAQDWVITK, and DEYGWEHSAVSEGAWTK. The second hit was the hypothetical protein AN4102.2 from *Aspergillus nidulans *FGSC A4 (gi|67527650), with a mass of 89450 Da; one peptide was matched to this protein: HYLLNEQEHFR.

### Cloning and sequence analysis of the β-glucosidase gene, *bgl*3

Degenerate primers were designed based on the peptide sequences obtained from the LC-MS/MS analysis to amplify a partial sequence of the β-glucosidase gene from *A. fumigatus *Z5. A 684-bp amplicon was obtained by PCR using the degenerate primer pair, BglF and BglR. To amplify the full-length of the *bgl3 *gene, primers (Table 2) were designed based on the determined 684-bp fragment. Tertiary PCR of 5' hiTAIL-PCR for bgl3 with the AD3 random primer and bgl3F primers resulted in a 953-bp fragment. For 3' hiTAIL-PCR of *bgl3*, the first round of tertiary PCR produced a 683-bp fragment with the bgl3R-a primers and the AD3 random primer. The forward primers (bgl3R-b) for the second round were designed based on the sequences obtained in the first round, and the AD4 random primer was used as the reverse primer. A 715-bp fragment was amplified in the second round of the hiTAIL-PCR. All of the PCR products were sequenced after purification and spliced using the DNAMAN 6.0 sequence analysis tool (Lynnon Biosoft, USA). Finally, PCR with primers bgl3-5' and bgl3-3' resulted in a 3,061-bp amplicon. The BLAST result indicated that the predicted sequences belonged to β-glucosidase. A 2622-bp fragment was cloned from the synthesized cDNA using the bgl3-5' and bgl3-3' primers. The alignment results between two sequences indicated that the *bgl*3 gene was interrupted by 8 introns.

**Table 2:** Primers used in the process of hiTAIL-PCR

AD and reactions	names	Sequences (5'-3')
Arbitrary degenerate primer	AD1	**ACGATGGACTCCAGAG***VNVNNNGGAA*

	AD2	**ACGATGGACTCCAGAG***BNBNNNGGTT*

	AD3	**ACGATGGACTCCAGAG***VVNVNNNCCAA*

	AD4	**ACGATGGACTCCAGAG***BDNBNNNCGGT*

	AC1	**ACGATGGACTCCAGAG**

The upstream hiTAIL-PCR	bgl3R-1a	CAACTCGTGCATGGTCTTGTC

	bgl3R-2a	**ACGATGGACTCCAGTC**CGTGATGTTGTAACCATATC

	bgl3R-3a	TGCTCCTGTTCATTCAGAATG

The downstream hiTAIL-PCR	bgl3F-1a	CAGCCTGGCGTGTCGATGACA

	bgl3F-2a	**ACGATGGACTCCAGTC**TCGTATCATGACCGCGTAC

	bgl3F-3a	CATTCTGCTGTCTCCGAGGGAG

	bgl3F-1b	ACGGCGAGGCCGTCATTGACACT

	bgl3F-2b	**ACGATGGACTCCAGTC**CGATTGTGGTTATTCACAGTG

	bgl3F-3b	CAACGTCACTGCCATCATCTG

	bgl3F-1c	CTCAACTCGACCGACCTCGA

	bgl3F-2c	**ACGATGGACTCCAGTC**CTACGGCTGGGAGGACTCG

	bgl3F-3c	TGGTAACCCTACCCTTTATCA

V (G/C/A), B (G/C/T), D (G/A/T), N (A/T/C/G)

**Figure 2:**
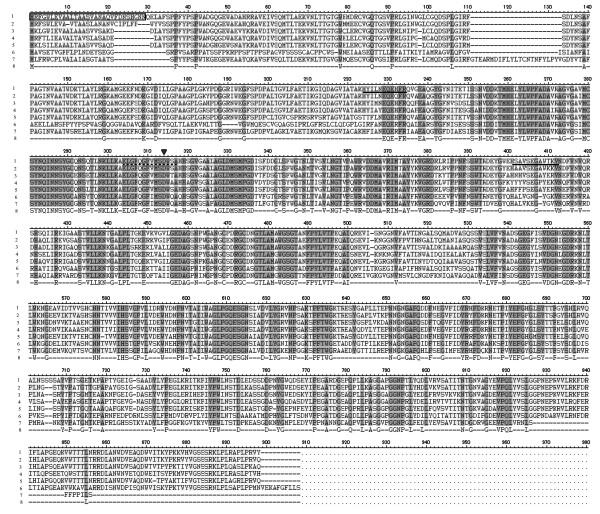
**Alignment of sequences deduced from *A. fumigatus *nBgl3 with other cellulases using BioEdit**. The sequences are numbered: 1 (*Aspergillus fumigatus *Z5 nBgl3), 2 (*Aspergillus clavatus *NRRL 1 putative β-glucosidase: XP_001269582.1), 3 (*Aspergillus flavus *NRRL3357 putative β-glucosidase: XP_002383240.1), 4 (*Aspergillus niger *β-glucosidase: CBA02054.1), 5 (*Aspergillus avenaceus *β-glucosidase: AAX39011.1), 6 (*Paracoccidioides brasiliensis *Pb01 β-glucosidase: XP_002795910.1), 7 (*Arthroderma benhamiae *CBS 112371 putative β-glucosidase: EFE35611.1), and 8 (consensus); the predicted signal peptide sequence is shown in the box. The conserved region used for the degenerate PCR primer is marked by a straight line, the active site is marked by a broken line, and the putative nucleophile in the catalytic center is marked by an inverse triangle.

Nucleotide sequence analysis showed that the sequence of *bgl*3 contained an open reading frame (ORF) of 2622 bp. The deduced protein sequence of *bgl*3 contained an N-terminal signal sequence of 29 amino acids, as predicted by the SignalP server system http://www.cbs.dtu.dk/services/SignalP/ (Figure 2). After removing the signal sequence, the mature peptide contained 844 amino acids with a predicted molecular mass of 91.47 kDa and had a theoretical pI of 5.01 http://web.expasy.org/compute_pi/. The deduced amino acid sequence of *bgl*3 was analyzed using the BLAST tool on NCBI, and the results indicated that the β-glucosidase cloned from *A. fumigatus *Z5 belongs to the GH3 family of enzymes. The amino acid sequence contained fifteen potential N-glycosylation sites as predicted by the NetNGlyc 1.0 server program http://www.cbs.dtu.dk/services/NetNGlyc/.

### Expression of the *bgl*3 gene in yeast and purification of the recombinant enzyme

**Figure 3:**
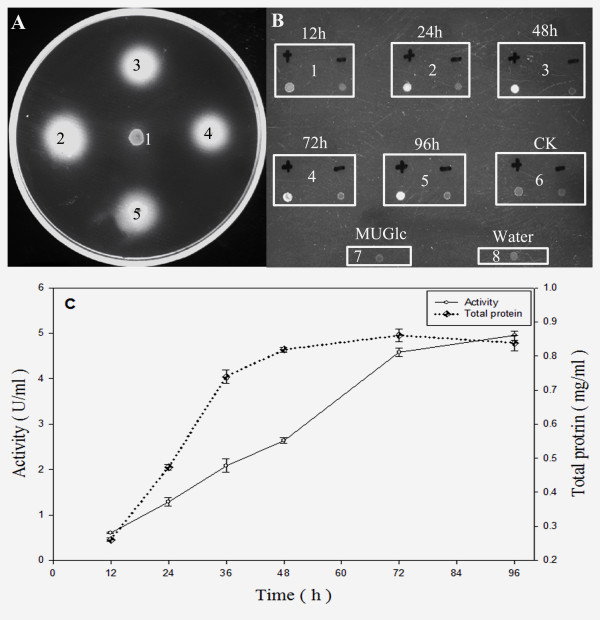
**Expression of the *bgl3 *gene in *P. pastoris *X33**. *P. pastoris *transformed with pPICZαA or pPICZαA/*bgl3*was spotted on a YPM plate and grown at 30°C for 2 days, and the β-glucosidase activity was detected with MUG as substrate. In Figure 3A, spot 1 indicated pPICZαA, and spots 2, 3, 4, 5 indicated pPICZαA/bgl3. In Figure 3B, frame 1 indicated the time point of 12 h, frame 2 indicated the time point of 24 h, frame 3 indicated the time point of 48 h, frame 4 indicated the time point of 72 h, frame 5 indicated the time point of 96 h, frame 6 indicated the crude extract from pPICZαA, frame 7 indicated the solution of MUGlc, frame 8 indicated ddH_2_O; "+", crude extract with MUGlc, "-", crude extract only.

The gene expression vector pPICZαA-*bgl3 *was constructed as described in the materials and methods section. In this construction, *bgl3 *fused with the α-factor signal sequence at its N-terminus was expressed under the control of the AOX1 promoter. The transformants resistant to 1000 μg ml^-1 ^Zeocin were chosen for expression on YPM plates. *P. pastoris *transformants of pPICZαA-*bgl3*showed strong luminescence around the colonies under UV light when MUG was used as substrate, but the negative control transformed with the pPICZαA vector did not show a clear zone (Figure 3A). The transformants with a large clear zone on the plates were cultured in liquid YPM and induced with methanol for 96 h; the supernatants were collected to determine protein concentration and β-glucosidase activity (Figure 3B and 3C). The transformants had the highest protein content (0.86 mg ml^-1^) after 72 h and the highest β-glucosidase activity (4.95 U ml^-1^) after 96 h under the methanol-induced conditions. However, no β-glucosidase activity was detected in the negative control culture, transformed with pPICZαA, when the same culture conditions were used (data not shown). The C-termini of the recombinant enzyme contained 6 × His-tags; therefore, the recombinant protein was purified with use of a Ni-NTA affinity column. After elution from the His-binding column, the rBgl3 protein was purified 3.5-fold with a 62.1% yield.

### SDS-PAGE, native PAGE and western blot analysis of the recombinant protein

**Figure 4:**
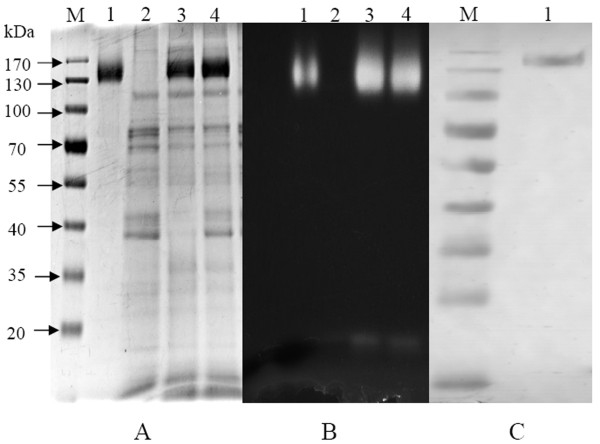
**SDS-PAGE (A), Native PAGE (B) and western blot analysis (C) of the recombinant Bgl3**. Lane M: molecular weight markers; Lane 1: purified recombinant Bgl3; Lane 2: culture supernatant of empty vector; Lane 3, 4: culture supernatant of recombinant Bgl3.

The presence of the recombinant protein was verified via SDS-PAGE analysis of the sample prepared as described in the material and methods section, and SDS-PAGE analysis indicated that a recombinant protein band of approximately 130 kDa (Figure 4A) was detected in the liquid medium of *P. pastoris *harboring pPICZαA/*bgl3*. For this sample, a final concentration of 1% methanol was used to induce protein expression. The purified protein was examined for its ability to hydrolyze MUG incorporated into the gel, and the native PAGE analysis showed that the purified rBgl3 was able to hydrolyze MUG, as clear bands were visualized under UV 365 nm after incubating the gels at 50°C for 5 min (Figure 4B1). However, no fluorescent band appeared in the lane of crude extract from *P. pastoris *harboring pPIZαA vector (Figure 4B2).

The Western blot results indicated that the purified Myc-tagged proteins were transferred onto the NC membrane successfully, and the purified Myc-tagged antibody migrated at approximately 130 kDa, corresponding to the monomeric form of the Myc-tagged rBgl3 fusion protein (Figure 4C). Moreover, SDS-PAGE analyses, zymograms and western blot analyses revealed that the protein purified from the liquid medium of *P. pastoris *transformed with pPICZαA/*bgl3* was the expected rBgl3.

### Effects of pH and temperature on the activity and stability of purified nBgl3 and rBgl3

**Figure 5:**
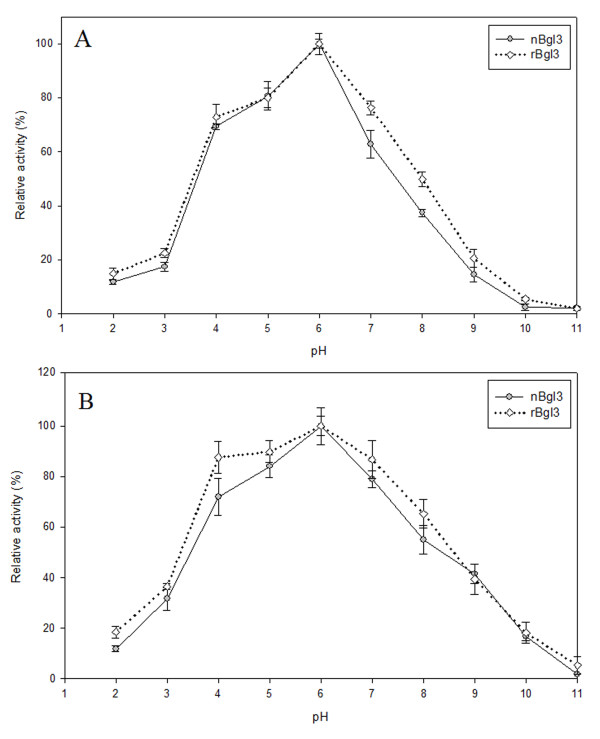
**Optimal pH (A) and pH stability (B) of purified nBgl3 and rBgl3**. Results are the average of three replicates, and bars indicate standard error of three replicates. The activity was detected by the standard method by changing the buffer to obtain the desired pH, and the buffers used were 0.1 M Gly-HCl Buffer (pH 2), 0.1 M Citric-NaOH (pH3-5), 0.1 M sodium phosphate (pH 6-8) and 0.1 M Gly-NaOH (pH9-11). For pH stability, the enzymes were incubated at 50°C for 20 min before measurement of the remained activity.

Both nBgl3 and rBgl3 were the most active toward pNPG at pH 6.0 (Figure 5A). The effects of different pH on the stability of purified nBgl3 and rBgl3 were monitored (Figure 5B). After incubation at 50°C for 20 min, both enzymes were fairly stable in the pH range of 4.0-7.0; over 80% of the original activity for rBgl3 and 60% of the original activity for nBgl3 were retained, indicating that both of the β-glucosidases were acidic cellulases. However, they were sensitive to pH below 4.0, as the activities decreased quickly.

**Figure 6:**
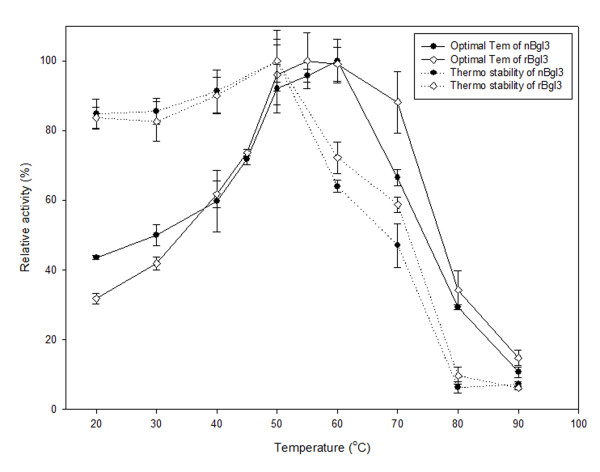
**Optimal temperature and thermostability of purified nBgl3 and rBgl3**. The optimal temperature was measured at different temperatures ranging from 20 to 90°C. Results are the average of three replicates, and bars indicate standard error of three replicates. For thermostability, the enzymes were incubated for 1 hour at different temperatures at pH 6.0 before measurement of the remained activity.

The effects of temperature on the activity and stability of two purified β-glucosidases are shown in Figure 6. nBgl3 and rBgl3 displayed maximal activity at 60°C. nBgl3 was moderately stable when incubated for one hour at temperatures up to 50°C and retained more than 50% of its activity at 70°C, while rBgl3 maintained more than 60% of the original activity at temperatures between 20 and 60°C for one hour, but it lost most of its activity when incubated at the temperatures between 60°C and 80°C.

### Effects of metal ions and reagents on the activities of purified nBgl3 and rBgl3

The effects of various metal ions and reagents on the activities of both β-glucosidases were evaluated, and the results are shown in Table 3. Their activities were strongly stimulated by 1 mM Ni^2+^, and 1 mM concentrations of Mg^2+^, Fe^2+^, Ca^2+^, Cd^2+^, or Pb^2+ ^slightly increased the enzyme activities. However, 1 mM concentrations of Fe^3+^, Li^+^, Co^2+^, Cu^2+^, Mn^2+^, Cr^3+^, or Hg^2+ ^inhibited the enzyme activities of nBgl3 and rBgl3. Hg^2+ ^was an effective inhibitor, reducing the enzyme activity of nBgl3 to 37.5% of the original value and that of nBgl3 to 35.0% of the original value. SDS, Triton X-100 and EDTA had no obvious effects on their activities.

**Table 3:** Effects of various metal ions, chemical agents and chelating agent on the activities of two purified β-glucosidases.

Effectors	Relative activity (%)	
	
	nBgl3	rBgl3
Control	100	100

metal ions(1 mM)		

Ni^2+^	130.5 ± 7.4^a^	121.4 ± 3.3

Ba^2+^	102.1 ± 3.8	96.5 ± 7.2

Fe^+2^	115.2 ± 1.7	107.5 ± 3.8

Fe^3+^	51.4 ± 3.1	55.6 ± 2.5

Li^+^	98.3 ± 2.7	94.1 ± 8.6

Ca^2+^	105.6 ± 7.6	101.8 ± 4.9

Co^2+^	93.7 ± 3.3	93.3 ± 8.5

Cu^2+^	65.8 ± 1.5	67.1 ± 3.7

Mg^2+^	137.1 ± 5.1	133.9 ± 8.2

Mn^2+^	94.6 ± 4.7	91.5 ± 7.6

Cr^3+^	75.9 ± 6.0	73.7 ± 5.1

Cd^2+^	102.6 ± 7.1	96.2 ± 4.7

Pb^2+^	115.2 ± 2.8	105.8 ± 6.1

Hg^2+^	37.5 ± 2.1	35.0 ± 1.6

Surfactants		

SDS (2 mg ml^-1^)	95.0 ± 2.4	93.2 ± 1.5

Triton X-100 (20 mg ml^-1^)	103.2 ± 5.8	109.7 ± 2.7

Chelating agent (10 mM)		

EDTA	90.1 ± 1.3	95.72 ± 7.3

Results are the mean of three replicates

nBgl3: the native Bgl3; rBgl3: the recombinant Bgl3; ^a^Standard deviations of relative activity

### Substrate specificity and kinetic parameters of purified nBgl3 and rBgl3

The specificities of nBgl3 and rBgl3 against various substrates are presented in Table 4. The results showed that both nBgl3 and rBgl3 were maximally active against pNPG, with specific activities of 103.5 ± 7.1 and 101.7 ± 5.2 U mg^-1^, respectively. Both β-glucosidases also hydrolyzed cellobiose effectively, resulting in specific activities of 64.1 ± 3.8 U mg^-1 ^for nBgl3 and 59.4 ± 2.1 U mg^-1 ^for rBgl3. The purified enzymes had very little or no activity on carboxymethyl cellulose (CMC), xylan, lichenan, laminarin or avicel substrates. Neither enzyme showed activity toward 4-nitrophenyl-α-D-glucopyranoside or lactose, which belong to the glycosyl group of α-glycosides.

**Table 4:** Hydrolytic specific activities of two purified β-glucosidases (nBgl3 and rBgl3) on various substrates.

Substrate	Specific activity (U mg^-1^)		Relative activity^b ^(%)		Linkage of glycosyl group
		
	nBgl3	rBgl3	nBgl3	rBgl3	
4-Nitrophenyl-β-D-glucopyranoside (1 mM)	103.5 ± 7.1^a^	101.7 ± 5.2	100	100	βGlc

4-Nitrophenyl-α-D-glucopyranoside (1 mM)	< 0.01	< 0.01	0	0	αGlc

Cellobiose (5 mM)	64.1 ± 3.8	59.4 ± 2.1	61.9	58.4	β (1,4) Glc

Cellotriose (5 mM)	41.4 ± 3.2	39.3 ± 1.2	40.0	38.6	β (1,4) Glc

Cellotetraose (5 mM)	35.5 ± 1.6	32.7 ± 2.3	34.3	32.2	β (1,4) Glc

Cellopentaose (5 mM)	29.5 ± 2.0	23.7 ± 1.3	28.5	23.3	β (1,4) Glc

Carboxymethyl cellulose (1%, w/v)	< 0.01	< 0.01	0	0	β (1,4) Glc

Lactose (1%, w/v)	< 0.01	< 0.01	0	0	α (1,4) Glc

Xylan (1%, w/v)	< 0.01	< 0.01	0	0	β (1,4) Xyl

Lichenan (1%, w/v)	13.2 ± 0.1	9.7 ± 0.2	12.8	9.5	β (1,3-1,4) Glc

Laminarin (1%, w/v)	18.4 ± 1.3	17.5 ± 0.6	17.8	17.2	β (1,3) Glc

Gentiobiose (1%, w/v)	34.3 ± 2.4	29.4 ± 1.6	33.1	28.9	β (1,6) Glc

Salicin (1%, w/v)	38.1 ± 1.5	31.8 ± 2.7	36.8	31.3	βGlc

Avicel (1%, w/v)	2.1 ± 0.1	1.7 ± 0.08	2.0	1.7	β (1,4) Glc

The purified enzyme was assayed in the standard assay condition with various compounds. Results are the mean of three replicates and varies from the mean by not more than 10%

^a^Standard deviations of specific activity; ^b^The activity for pNPG was defined as 100%; nBgl3: the native Bgl3; rBgl3: the recombinant Bgl3

The kinetic parameters of two purified β-glucosidases were determined by applying a nonlinear curve fit, and the results are shown in Table 5. The Km values of nBgl3 and rBgl3 for pNPG were 1.73 mM and 1.76 mM, respectively, and the Km values of nBgl3 and rBgl3 for cellobiose were 1.75 mM and 2.20 mM, respectively. The Vmax values obtained for pNPG and cellobiose by nBgl3 under standard assay condition were 141.60 and 52.3 μmol min^-1 ^mg^-1^, respectively; the Vmax values of rBgl3 toward these substrates were lower than those of nBgl3. The catalytic efficiencies of the two purified β-glucosidases, given by the kcat/Km ratios, were much higher for pNPG than for cellobiose.

**Table 5:** Kinetic parameters of two purified β-glucosidases

Substrates	Enzyme	Vmax (μmol min^-1 ^mg^-1^)	Km (mM)	Kcat (s^-1^)	Kcat/Km (s^-1 ^mM^-1^)
pNPG	nBgl3	141.60 ± 2.61	1.73 ± 0.19	217.12	125.50

	rBgl3	131.40 ± 2.21	1.76 ± 0.18	284.70	161.76

Cellobiose	nBgl3	52.37 ± 0.87	1.75 ± 0.17	80.30	45.89

	rBgl3	52.89 ± 1.12	2.20 ± 0.26	114.60	52.09

## Discussion

In this study, a β-glucosidase (nBgl3) from *A. fumigatus *Z5 was purified to homogeneity. The purification protocol that we followed involved four steps: ammonium sulfate precipitation, cation exchange, gel filtration, and affinity chromatography. In the affinity chromatography step, microcrystalline cellulose was used as a column agent to purify nBgl3, and a high purification fold was obtained after this step. A common problem during the β-glucosidase purification process is that it usually requires complicated steps with a combination of various chromatographic columns [21]. Kim et al., [22] studied the adsorption kinetics and behaviors of cellulase components on microcrystalline cellulose, and their results indicated that microcrystalline cellulose had a high affinity for different cellulolytic glucosidases. Thus, microcrystalline cellulose chromatography is a suitable method for β-glucosidase purification and could greatly simplify and reduce the cost of the purification process.

Based on the internal amino acid sequence of nBgl3, the encoding gene *bgl*3 was cloned by hiTAIL-PCR. The *bgl3 *gene of *A. fumigatus *Z5 is not a novel gene because it is similar to some sequences submitted to GenBank. We thoroughly searched the databases and did not find any information about cloning, expression, or characterization of this gene from *Aspergillus fumigatus*. Thus, this study is the first report on the purification, expression and characterization of this enzyme in *Aspergillus fumigatus*. Glycosidases have been classified into several families based on amino acid sequence similarities, and most known BGLs belong to either family 1 or family 3. An NCBI BLAST search of the deduced amino acid sequence of *bgl*3 indicated that the nBgl3 from *A. fumigatus *Z5 belongs to the GH3 family. Most cellulases generally contain two or more discrete domains: a catalytic domain and a highly conserved cellulose-binding domain, with an often-glycosylated hinge connecting these two domains [23]. The highest sequence identity was obtained when compared with other related enzymes (Figure 2), and a conserved catalytic domain sequence (ELGFQGFVMSDWSA) was found in most of the enzymes. *P. purpurogenum *BGL, which was purified by Jeya et al. [23], contained a conserved GFVMTD sequence. These residues were identified as part of the catalytic domain of GH3 family proteins; the aspartic acid residue has been shown to be highly conserved and confirmed to be the active-site residue of BGLs in GH3 enzymes [24].

Several GH3 β-glucosidases have been purified from fungi or successfully expressed in yeast [25-27]. However, except for studies by Jeya et al. [23] and Hong et al. [28], few reports about the purification of β-glucosidase from thermophilic fungi and the functional expression of a thermostable β-glucosidase gene in yeast have been published. Thermostability is an important property of β-glucosidase during enzymatic hydrolysis, which converts cellobiose to reducing sugars. Steam is usually applied to make the biomass more easily degraded, especially during the saccharification step. A thermostable β-glucosidase, combined with other thermostable enzymes, could be used directly after the heating step without a pre-cooling process, thereby decreasing the processing time, saving energy, reducing the risk of contamination, and improving fermentation yields and qualities [14]. Both nBgl3 and rBgl3 are thermostable β-glucosidases; nBgl3 retained more than 40% of its peak activity, and rBgl3 retained more than 50% of its peak activity at 70°C for one hour. Thus, both of two β-glucosidases could be used in various fields, such as bioenergy production and food processing. The demand for thermostable β-glucosidase is rapidly increasing and has become the driving force for studies on a wide range of sources. However, the low yield of β-glucosidase and the high viscosity of the induction media have limited the scale-up for the production of this enzyme at an industrial scale [29]. The rapid developments of molecular biology make it possible to express active enzymes in yeast for large-scale production [14]. Protein production in yeast has the advantages of ease of handling, rapid growth, and highly efficient transformation Hong et al. [28].

β-glucosidases are divided into three types based on substrate specificity: aryl-β-glucosidases that have strong affinities for aryl-β-glucosides, cellobiases that hydrolyze only oligosaccharides, and broad-specificity β-glucosidases that exhibit activity toward many substrate types [30]. In our study, various substrates belonging to different glycosyl groups were used to detect the specific activities of purified nBgl3 and rBgl3. Our results indicated that both β-glucosidases were broad-specificity types, as both can hydrolyze a range of (1,3)-, (1,4)-, and (1,6)-β-diglycosides. β-glucosidases with very broad specificity have been purified from different sources, especially from fungi [9,12,18,26]. However, except for *Sulfolobus shibatae *[31], few strains can secrete β-glucosidases that can hydrolyze both β- and α-glucosides. Neither of the β-glucosidases that we purified exhibited activity toward 4-Nitrophenyl-α-D-glucopyranoside or lactose, both of which belong to the glycosyl group of α-glycosides. However, both purified enzymes exhibited activity toward cellooligosaccharides in a similar manner in which catalytic efficiency decreased as the number of glucose units increased as HGT-BG [19], indicating that these enzymes possess some exoglucanase activities.

Various metal ions and other agents modified the activity of the purified enzyme, and the effects on the purified enzymes must be investigated in detail because many catalytic processes require their addition. In our study, Fe^3+^, Li^+^, Co^2+^, Cu^2+^, Mn^2+^, Cr^3+^, and Hg^2+ ^inhibited the enzyme activities of nBgl3 and rBgl3. Fe^3+^, Cu^2+ ^and Hg^2+ ^also inhibit HGT-BG [19] and BglA [32], suggesting that the active catalytic sites of these enzymes might posses thiol groups that cause sensitivity to inhibition by Hg^2+^.

## Conclusions

This report is the first on the purification, expression and characterization of this enzyme from *A. fumigatus*. In this study, an extracellular enzyme nBgl3 was purified to electrophoretic homogeneity from the crude extract of *A. fumigatus *Z5. The gene *bgl*3 was cloned based on the internal sequences of nBgl3 obtained from the LC-MS/MS results. Sequence analysis indicated that nBgl3 is a member of the GH3 family of enzymes. Several biochemical properties of purified nBgl3 and rBgl3 were analyzed, including specific activity, pH stability, thermostability, and kinetic properties (Km and Vmax).

### Experimental procedures

#### Organisms plural

*A. fumigatus *Z5 was isolated and identified as previously reported [33]. Potato glucose agar (PDA) was used for the cultivation of *A. fumigatus *Z5; the liquid medium used for cellulase production was composed of the following: 10 g pure ball-milled cellulose powder (Sigma, USA), 1 g KH_2_PO_4_, 0.5 g urea, 0.5 g (NH_4_)_2_SO_4_, 0.5 g MgSO_4_·7H_2_0, 7.5 mg FeSO_4_·7H_2_0, 2.5 mg MnSO_4_·H_2_O, 3.6 mg ZnSO_4_·7H_2_O, 3.7 mg CoC1_2_·6H_2_O, and 0.5 g CaC1_2 _in 1000 ml of water. The culture was incubated at 50°C for an appropriate period.

*P. pastoris *X33 (Invitrogen) was used to express the *bgl3 *gene, and *Escherichia coli *Top10 (stored in our lab) was used for plasmid construction. YPD medium (1% yeast extract, 2% peptone, and 2% glucose, pH 6.0) was prepared according to the *Pichia *expression system manual from Invitrogen and was used to propagate the rBgl3 recombinant protein. YPM medium (1% yeast extract, 2% peptone, and 1% methanol, pH 6.0) was used as the induction medium.

### Purification of nBgl3 secreted by *A. fumigatus *Z5

β-glucosidase was purified according to methods used by Yan et al., [21] and Daroit et al., [25], with some modifications. After the incubation period, the culture was filtered and centrifuged (12,000 rpm for 10 min). The supernatant was utilized as crude enzyme for the purification process. Protein extraction was performed by ammonium sulfate precipitation according to Liu et al. [14].

The crude enzyme was loaded onto a Q-sepharose FF (Amersham Biosciences) column (1.6 × 10 cm) that had been previously equilibrated with sodium phosphate buffer (10 mM, pH 6.0). After the columns were washed with four column volumes of the same buffer, a linear gradient elution of 0 to 0.3 M sodium chloride was performed, and the fractions with β-glucosidase activity were pooled, completely dialyzed against 10 mM sodium acetate buffer (pH 5.0), and freeze-dried to an appropriate volume. The proteins were loaded onto a Sephadex G-100 gel filtration column (1.6 × 60 cm) (Amersham Biosciences) and flowed at a rate of 25 ml h^-1^. The proteins were eluted off the column with sodium phosphate buffer (20 mM, pH 6.0), and 80 fractions of 1 ml were collected. For each fraction, β-glucosidase activity was assessed, and absorbance at 280 nm was used to monitor the protein content in the column fractions. The fractions with enzyme activity were pooled, freeze-dried, and re-dissolved in an appropriate volume of sodium acetate buffer.

The microcrystalline cellulose (MC) column was prepared as follows: the MC powders were suspended in 10 mM sodium acetate buffer (pH 5.0) containing 0.8 M sodium chloride, stirred on a magnetic stirrer for 2 h, and then packed into a glass column. The column (2.5 × 18 cm) was washed with 4 column volumes of the same buffer and then equilibrated with 10 mM sodium acetate buffer (pH 5.0). The fractions containing active enzyme were applied to an MC column and washed stepwise with 0.1, 0.2, 0.3, 0.4, and 0.5 M sodium chloride in 10 mM sodium acetate buffer (pH 5.0), and the fractions with β-glucosidase activity were collected.

### Internal amino acid sequence of nBgl3 by LC-MS/MS

The purified β-glucosidase was analyzed by LC-MS/MS for internal amino acid sequences according to Shevchenko et al., [34]. To identify the protein sequence, a homology search method was employed using the MS data analysis program MASCOT http://www.matrixscience.com/, a powerful search engine that uses mass spectrometry data to identify proteins from primary sequence databases. The partial amino acid sequence was used to identify analogous proteins through a BLAST search of the nonredundant protein database.

### Isolation of genomic DNA and mRNA, and synthesis of cDNA

Genomic DNA of *A. fumigatus *Z5 was extracted as described by Moller et al. [35]. After induction by cellulose for approximately 4 days, the mycelium was collected for the mRNA extraction. The mRNA isolation was performed using the E.Z.N.A.™ Fungal RNA Kit (Omega Bio-tek, Inc. R6840-01). cDNA synthesis and reverse transcriptase (RT) reactions were performed on the mRNA using the RevertAid™ First Strand cDNA synthesis kit (Fermentas, #K1621).

### Cloning of the β-glucosidase gene, *bgl*3

The degenerate primers BglF (CATTACWTHCTNAATGAACAGGAGC) and BglR (CACHTTGGTCCAKGCTNCCT) were designed based on the partial peptide sequences (HYILNEQEHFR and HSAVSEGAWTKV) obtained from the LC-MS/MS sequencing. The PCR was performed using the following mix: 2.5 μL 10 × PCR buffer, 2.5 μL Mg^2 ±^, 2 μL of 10 mM dNTPs, 10 pmol/μL of each primer, and 0.5 U of Taq DNA polymerase in a total volume of 25 μL (Takara, Dalian, China). The amplification was performed using a thermal cycler (Bio-Rad S1000, USA) with the following cycling parameters: an initial denaturation step at 95°C for 5 min, 30 cycles of amplification (denaturation at 94°C for 30 s, annealing at 50-58°C for 30 s, extension at 72°C for 30 s), and a final elongation step at 72°C for 10 min. For analysis, 2 μL of the reaction mixture was electrophoresed on a 1% agarose gel and stained with an ethidium bromide solution (5 μg ml^-1^).

After DNA sequencing, a partial DNA sequence was identified. To obtain the 5'-end and 3'-end of the β-glucosidase fragments, hiTAIL-PCR was applied according to Liu et al. [36]. The primers used in the hiTAIL-PCR are shown in Table 1, and genomic DNA was used as the template. The PCR product was purified and cloned into the PMD19-T vector (TaKaRa, Dalian, China), and its nucleotide sequence was subsequently determined. By aligning the sequences of the 5'-end and 3'-end PCR products, the full-length cDNA sequences of β-glucosidase were deduced and obtained through RT-PCR using the following specific primers: bgl3-5' (ATGAGATTCGGTTGGCTCGAGGTGG) and bgl3-3' (CTAGTAGACACGGGGCAGAGGCGCT). The full-length products were purified, ligated into the pMD^®^19-T Vector (Takara, Dalian, China), and transformed into competent *E. coli *Top10 cells. The full-length *bgl3 *gene was confirmed by sequencing, and the recombinant plasmid was designated as PMD - *bgl3*.

### Construction of the expression plasmid

The open reading frame (ORF) of the *bgl*3 gene, excluding the native signal sequence (amino acids 1-29), was amplified by PCR using the primers bgl3E-5' (ATAAGAAT**GCGGCCGC**CAGGAATTGGCTTTCTCTCCAC) and bgl3E-3' (GC**TCTAGA**TAGTAGACACGGGGCAGAGG). The recombinant plasmid PMD-bgl3 was used as template. *Not *I and *Xba *I sites were introduced into the bgl3E-5' and bgl3E-3' primers, respectively (underlined). After double digestion with *Not *I and *Xba *I, the PCR product was inserted into the vector pPICZαA (Invitrogen, USA). Proper construction was confirmed by restriction digestion and DNA sequencing. This construct was designated as pPICZαA/*bgl3*.

The recombinant plasmid pPICZαA-*bgl3* was linearized with *Sac *I (TaKaRa, Dalian, China) before introduction into *P. pastoris *X33 by electroporation (Gene Pulser Xcell™ Electroporation System #165-2660, Bio-Rad, USA). The cells were pulsed using the following parameters: 1.5 kV, 200 μF, and 200Ω. The transformants were screened by selection on YPDS (1% yeast extract, 2% peptone, 2% dextrose, 1 M sorbitol, 2% agar) plates containing Zeocin™ at a final concentration of 1000 μg mL^-1 ^(Zeo1000 plates) according to Chen et al. [37]. *P. pastoris *X-33 transformed with the vector pPICZαA was used as a control.

### Expression and purification of rBgl3 from *Pichia pastoris *X33

Colonies from the Zeo1000 plates were inoculated onto a YPM plate to induce the expression of the β-glucosidase gene, as was previously described by Hong et al. [28], with some modifications. The transformants were cultivated on YPM solid medium for 2 days, and then the plates were overlaid with 0.8% agar containing 5 mM MUG (β-4-methylumbelliferyl-β-D-glucose). Detection of β-glucosidase activity was performed by exposing the plate to UV light (365 nm) to detect luminescence after incubation for 10 min at 50°C.

Transformants with the strongest β-glucosidase activity on the YPM plate were cultured at 30°C for 96 h in 100 ml of YPM liquid medium after a 24 h preculture in 200 ml of YPD medium. The supernatant was then recovered by centrifugation and subjected to precipitation at 80% ammonium sulfate saturation. After dialysis against 50 mM sodium phosphate buffer (pH 6.8), the protein was collected and stored at -20°C. The expressed His_6_-tagged proteins were purified with Ni-NTA Sepharose (QIAGEN) according to Kabir et al. [38].

### SDS-PAGE, native PAGE and Western blot analysis of rBgl3

SDS-PAGE was performed with a 10% (w/v) polyacrylamide gel in accordance to the method described by Laemmli [39]. The crude enzyme samples mixed with the same volume of loading buffer were boiled at 100°C for 4 min and then subjected to SDS-PAGE. The gel was stained with Coomassie Brilliant Blue R-250 and destained with destaining solution (2.5% methanol, 10% acetic acid) by shaking at 100 rpm min^-1 ^for 1-2 h.

Detection the in-gel β-glucacosidase activity was performed by native PAGE using 10% and 5% polyacrylamide as the separation and stacking gels, respectively [40]. Electrophoresis was run at a constant current of 20 mA per slab at 4°C for 3 h, and Tris-glycine buffer (pH 8.3) was used as the electrode buffer. After electrophoresis, the gels were washed with distilled water and then overlaid with 0.5 mM 4-methylumbelliferyl β-D-glucopyranoside (Sigma, USA) in 0.1 M succinate buffer (pH 5.8); the presence of a fluorescent reaction product was visualized under UV light (365 nm) after incubating the gels at 50°C for 5 min.

The purified protein was separated on 12% polyacrylamide gels and was used for Western blotting. For Western blotting, SDS-PAGE-separated proteins were blotted onto a NC membrane with ECL Semi-dry Blotters (TE 70 PWR semi-dry transfer unit, Amersham) according to the manufacturer's specifications. After the membranes were blocked with blotting buffer (25 mM Tris, 192 mM glycine, 15% v/v methanol, pH 8.3, 1 L), the separated proteins were detected with an anti-Myc mouse antibody (Beyotime, China) and an anti-mouse IgG (H + L)-AP conjugate goat antibody (Beyotime, China). The BCIP/NBT Alkaline Phosphatase Color Development Kit (Beyotime, China) was used for detecting alkaline phosphatase. A broad-range protein marker (Fermentas, China) was used as a molecular weight marker.

### Enzyme assay and protein determination

β-glucosidase activity was determined by a microtitre plate method that measures the hydrolysis of p-nitrophenyl-β-D-glucopyranoside (pNPG) (Sigma, USA), as described by Parry et al. [41], with some modifications. A 25 μl portion of the culture filtrate or 25 μl of appropriately diluted purified enzyme was mixed with 25 μl of 200 mM sodium acetate buffer (pH 5.0) and 25 μl of distilled water and pre-incubated at 50°C for 5 min. The reaction was initiated by adding 25 μl of 10 mM pNPG; after incubation at 50°C for 10 min, the reaction was terminated by adding 100 μl of ice-cold 0.25 M Na_2_CO_3_. The developed color was read at 405 nm by Multi-Detection Microplate Readers (Spectra max M5, Molecular Devices) and translated to μmol of *p*-nitrophenol (pNP) using a standard graph prepared under the same conditions. One unit of β-glucosidase activity is expressed as the amount of enzyme required to release 1 μmol of pNP per minute under the above assay conditions. The activity was also examined with other substrates by measuring the amount of reducing glucose, according to Lin et al. [27]. β-Glucosidase activity on polysaccharides was determined at 50°C for 10 min and by measuring the reducing sugars according to the dinitrosalicylic acid (DNS) method [42]. Determination of the total protein in the supernatant was performed according to methods used by Branford [43], and bovine serum albumin (BSA) was used as a standard. Specific activity was expressed as units per milligram of protein.

### Characterization of nBgl3 and rBgl3

The pH profiles of nBgl3 and rBgl3 were constructed by determining activity toward pNPG at 50°C, as described above, in one of the following buffers: 50 mM citrate buffer (pH 3.0-6.0), 50 mM sodium phosphate (pH 6.0-8.0), 50 mM Tris-HCl (pH 8.0-9.0) and 50 mM glycine-NaOH (pH 9.0-11.0),. The effect of temperature on β-glucosidase activity was analyzed by measuring the enzyme activity, as described above, at various temperatures (20 to 90°C) in the 50 mM citrate buffer (pH 5.0).

The pH stabilities of nBgl3 and rBgl3 were assessed by incubating 100 μL of the purified nBgl3 or rBgl3 at 4°C for 24 h in 0.5 ml of various buffers adjusted to different pH values, followed by checking the remaining activity as described above. The buffers used were as follows: 0.1 M Gly-HCl Buffer (pH 2), 0.1 M Citric-NaOH (pH 3-5), 0.1 M sodium phosphate (pH 6-8) and 0.1 M Gly-NaOH (pH 9-11). The thermostability of the purified nBgl3 and rBgl3 was investigated by incubating the enzyme solutions in 50 mM sodium acetate buffer (pH 5.0) for 1 h, with temperatures ranging from 20 to 90°C. Subsequently, the remaining β-glucosidase activity of each treatment group was measured as described above by incubating the sample with 10 mM pNPG solution at 50°C for 20 min.

The inhibitory effect of various metal ions and EDTA on the activities nBgl3 and rBgl3 was determined according to Ding et al. [44], with some modifications. pNPG was used as a substrate in reaction mixtures containing 1 ml of 0.1 M sodium acetate buffer (pH 5.0), 0.5 ml 10 mM pNPG solution, 10 μl of purified enzymes and 1 mM inhibitor for 24 h at 4°C, and the remaining β-glucosidase activity was assessed as described above.

### Determination of kinetic parameters

Determination of the kinetic parameters (Vmax and Km) of hydrolysis of pNPG and cellobiose by the purified enzymes were determined at pH 5.0 and 50°C, and the values for Km and Vmax were estimated by applying a nonlinear curve fit using GraphPad Prism v5.01 from GraphPad Software (San Diego, CA). Catalytic constants (kcat) and catalytic efficiency ratios (Kcat/Km) were determined from the obtained kinetic parameter values.

### Accession Number

The nucleotide sequence of *bgl*3 was deposited into GeneBank under accession number HQ836475.

## Competing interests

The authors declare that they have no competing interests.

## Authors' contributions

LD performed the majority of the experiments and wrote the manuscript. RZ, ZZ and Miao did the MS, cloning and the Western blot, respectively. YX and SS studied the parameters of two enzymes. SQ was the corresponding author, he supervised the work and contributed to the manuscript. All authors have seen the manuscript and approved to submit to this journal.
